# Role of HLA-B Alleles and Clinical Presentation of B27 Negative Spondyloarthritis Patients from Mumbai, Western India

**DOI:** 10.1155/2014/327315

**Published:** 2014-03-06

**Authors:** Devaraj J. Parasannanavar, Anjali Rajadhyaksha, Kanjaksha Ghosh

**Affiliations:** ^1^Microbiology and Immunology Division, National Institute of Nutrition (ICMR), Jamai-Osmania, Tarnaka, Hyderabad, Andhra Pradesh 500007, India; ^2^Department of Medicine, King Edward Memorial Hospital, Parel, Mumbai 400 012, India; ^3^National Institute of Immunohaematology (ICMR), King Edward Memorial Hospital, 13th Floor, N.M.S. Building, Parel, Mumbai 400 012, India

## Abstract

Seronegative spondyloarthritis (SpA) are variably associated with HLA-B∗27 antigen. HLA-B∗27 negative SpA has also been reported from different parts of the world. There is paucity of data on this entity from Indian subcontinent. We studied 100 consecutively diagnosed HLA-B27 negative spondyloarthritis patients from a tertiary care center in India. Modified New York Criteria for ankylosing spondylitis (AS) and ESSG criteria for SpA were used for diagnosing patients. HLA-B∗27 typing was done by an in-house PCR-SSP technique in SpA patients to exclude B∗27 positive patients and PCR-SSOP technique was used to type 100 B∗27 negative SpA patients and 100 controls from the same ethnicity. Frequency of B∗07 was significantly increased (B∗07: % PF 54 versus 18; OR 5.348; 95% CI 2.808–10.186; *P* value 1.14*E* − 07), whereas frequency of B∗40 was significantly decreased (B∗40: % PF 17 versus 32; OR 0.435; 95% CI 0.222–0.850; *P* value 0.013) when compared with B∗27 negative controls. Among 100 SpA patients, 47 were undifferentiated spondyloarthritis and 33 patients were reactive arthritis patients. 40% of the patients were suffering from polyarticular arthritis, 35% had pauciarticular arthritis with knee joint, hip joint, ankle joint, and SI joint involvement. We conclude that B∗07 was significantly associated with B27 negative spondyloarthropathy from Western India and majority of B∗27 negative patients were uSpA.

## 1. Introduction

The spondyloarthritis (SpA) is a group of diseases which include mainly ankylosing spondylitis (AS), Reiter's syndrome/reactive arthritis (RS/ReA), enteropathic spondylitis (Crohn's disease and ulcerative colitis), Psoriatic Arthropathy (PsA), and undifferentiated spondylitis (uSpA). AS is a chronic inflammatory disease that begins primarily in the sacroiliac joints and goes on to involve the spine and other large joints. Reactive arthritis (ReA) is an acute nonporulant arthritis complicating an infection elsewhere in the body, usually genitourinary infection with* Chlamydia trachomatis* and enteritis due to gram negative enterobacteria such as* Shigella, Salmonella, Yersinia,* or* Campylobacter* species [1]. Psoriatic arthritis (PsA) refers to an inflammatory arthritis that typically occurs in individuals with Psoriasis [2]. Many patients, usually young adults, present with some features of one or more of the spondyloarthritis but lack criteria for these diagnosis; for example, a patient may present with inflammatory synovitis of one knee, achilles tendonitis, and dactylitis of one digit or sacroiliitis in the absence of other criteria for AS. Such patients are said to have undifferentiated spondyloarthritis (uSpA).

An association between HLA-B27 and AS was first reported in 1973 [3] and this was confirmed later [4–8]. The frequency of HLA-B27 ranges from 88 to 90% among patients as compared to 4–8% in controls. However, different races show different rates of association. The frequency of HLA-B27 with AS or other related SpA among Indian population varies from 30 to 94% as compared to 1.4–8% of the general population [8]. However, a more number of B27 negative individuals could develop SpA with typical clinical and radiological findings. There are reports which reveal that other HLA-B locus alleles have also be involved in B27 negative SpA patients from India [9] and worldwide [10–21]. Association of HLA-B7 CREG antigens (B7, B22, B27, B40, and B42) with AS patients among American Blacks was studied by Khan et al. [10, 11]. Subsequently these findings were confirmed among ReA and uSpA patients from different parts of the world [12–15]. The association of HLA-B40 with AS was reported by Robinson et al. for the first time, who found that HLA-B60 (Split of B40) was increased in B27 positive AS patients [16]. Later these findings were confirmed by other investigators among B27 positive as well as negative SpA patients [14, 16–18]. Earlier studies also showed the association between HLA-B16 (currently B38 and B39) and HLA-B35CREG (B18, B35, B51, and B62) with HLA-B27 negative Caucasian, Japanese, and German AS patients [19–21]. Hence, our aim was to study the role of HLA-B alleles and their clinical presentation in B*27 negative SpA patients from Western India.

## 2. Materials and Methods

### 2.1. Selection Criteria for Patients and Controls

In the present study, a total of 100 consecutive B*27 negative SpA patients were selected according to the revised New York criteria for AS [22] and ESSG for SpA [23]. Patients were evaluated by rheumatologist at K.E.M. and various hospitals from Mumbai and reevaluated by one of us (K.G). As per the selection criteria, SpA patients fulfilled at least any 4 of the following criteria: (1) insidious onset, (2) duration > 3 months, (3) radiological bilateral or unilateral sacroiliitis, (4) limitation of motion of the lumbar spine and chest expansion, and (5) improvement of morning stiffness with exercise and not relieved by rest. All patients were negative for B*27 typed by in-house PCR-SSP to exclude B*27 positivity [8]. These patients had undergone various tests including radiology of affected joints, sacroiliac joints, lumbar spine, cervical spine, full blood count with ESR, rheumatoid factor, anticitrullinated antibody (anti-CCP), and other autoimmune parameters like ANA and ds DNA. The study was carried out over a period of three years from April 2009 to March 2011. The study was approved by Ethics Committees of the institute. 100 age- and sex-matched healthy individuals which were negative for B*27 belonging to the same socioeconomic status and ethnic background during the same period comprised the controls for this study.

### 2.2. HLA Typing

#### 2.2.1. Molecular Typing

Genomic DNA extracted standard phenol-chloroform-isoamyl alcohol method from 5 mL of EDTA blood. B27 negative patients and controls were typed for B locus by polymerase chain reaction reverse line strip sequence-specific oligonucleotide hybridization (PCR-RLS-SSOP) strips (Dynal-Reli-SSO typing). Each strip for HLA-B typing carried a total of 62 immobilized SSOs. Genomic DNA was amplified using HLA-B locus specific biotinylated primers and hybridized with the SSO strips. Streptavidin-horseradish peroxidase (SA-HRP) conjugates for positive color development using hydrogen peroxide (H_2_O_2_) and tetramethylbenzidine (TMB) as substrate. The alleles were determined using the pattern interpretation software (PMP) supplied with the kit [24].

### 2.3. Statistical Analysis

The allele frequencies, odds ratio, 95% confidence interval, and chi-square with Yates' correction were estimated using SPSS software.

## 3. Results

Rheumatoid factor, anti-CCP antibody, ANA, and ds DNA were negative for all patients. Distribution of HLA-B alleles among 100 B*27 negative SpA patients and 100 controls is as shown in Table 1. The frequency of HLA-B*07 was significantly increased among patients as compared to the controls (B*07: %PF** 54** versus** 18;** OR** 5.348;** 95% CI** 2.808**–**10.186;** *χ* ^2^Y** 26.584;** *P* value** 1.14** *E* −** 07**), whereas frequency of B*40 was significantly decreased (B*40**: %**PF** 17** versus** 32;** OR** 0.435;** 95% CI** 0.222–0.850**; *χ* ^2^Y** 5.298**; *P* value** 0.013**). Clinical presentation of B*27 negative SpA patients is as shown in Table 2; when 52% of patients who are analyzed had the onset of clinical symptom between the age of 16 to 30 years, 30% showed the onset between the age between 31 to 45 years of age and male to female ratio of 4 : 1. Subgroup classification among B*27 negative SpA patients revealed that 47% patients had undifferentiated spondyloarthritis, 33% were ReA, 14% were AS, and JSpA were 4%. Analysis of the type of arthritis showed that 40% of the patients were suffering from polyarticular arthritis, 35% had pauciarticular arthritis, and 25% had monoarticular. Further analysis of the involvent of joints showed that 54% had knee joints affected, 46% had hip joint pain, 30% had ankle joints, and 25% of patients had SI joints involvement. When Scober's test results were analyzed, 50% of the patients had restricted spine flexion and 25% had diminished chest expansion. Enthesitis was present in 84% of the patients.

## 4. Discussion

The association between HLA-B27 with AS and related arthropathies has been known for a long time. But it is thought that in addition to B27, other HLA-B and DR alleles may increase susceptibility to the development of AS and related arthropathies [16]. The present study revealed the role of other HLA-B locus alleles and their clinical presentation among B27 negative SpA patients from Western India. In this study, HLA-B*07 was significantly increased among B27 negative SpA patients from Western India, which confirms earlier reports [9–15]. B*07 may be associated with AS either directly or because of linkage disequilibrium with further MHC gene. Our findings suggest that in addition to HLA-B*27, B*07 may also play a significant role in the development of SpA in Western Indian population.

Several studies have suggested that association of HLA-B*40 with B*27 positive as well as negative AS patients from various populations [16–18]. However, in our study B*40 was decreased among patients when compared with controls. An earlier study reported that even though B*40 frequency was increased marginally, no association was found between B*40 and SpA from north Indian population [24]. As HLA-B*27 and B*07 are more strongly associated with the disease, hence in our cohort of SpA patients where HLA-B*27 and B*07 are present in significant numbers, impact of B*40 has become statistically less. However, there are quite a few studies where B*40 was found to have stronger association with rheumatoid arthritis than with SpA [25], and these patients have higher tendency to have an associated pulmonary involvement. In our cohort such patients who were rheumatoid factor positive or were diagnosed with rheumatoid arthritis were meticulously excluded providing another reason for lower B*40 prevalence in our series.

Spondyloarthritis patients presented, either singly or in combination, with sacroiliitis with spondylitis; polyarticular arthritis; persistent pain and tenderness in the tendo-Achillis or heel; or pauciarticular arthritis associated with recent history (three months previously) of dysentery, together with high (ESR > 40 mm/h) and negative autoimmune parameters (ANA, dsDNA, and ANCA). The disease lasted for more than three months and responded to nonsteroidal anti-inflammatory drugs (NSAIDS) even before the diagnosis. Some patients showed systemic symptoms such as weight loss and low grade fever, 5 patients had uveitis, 4 patients had urethritis, and 2 patients had psoriatic arthropathies. Diseases at different duration revealed that majority of the patients were between age 16 to 30 years group in all durations. Chest expansion of ≤5 cm was present alone or together in 64% of the patients studied. Hence even in HLA-B*27 negative SpA patients, and this test is clinically useful. Undifferentiated form of SpA (47%), enthesitis (84%), and polyarticular arthritis was significantly over represented than in B*27 associated SpA as reported in the literature [15]. Hence HLA-B*27 negative SpA resembles classical HLA-B*27 positive variety of SpA in showing male preponderance and seronegativity (for both rheumatoid factor and anti-CCP antibodies). However, it differs substantially in clinical presentation by older age of presentation, high frequency of enthesitis, and polyarticular arthritis, but much lower frequency of sacroiliitis which corroborates earlier study on American pediatric SpA patients suggests that clinician needs look closely for enthesitis to diagnose SpA [26]. In our study HLA-B*40 was significantly underrepresented in our patients when compared to other reported studies elsewhere in the world [14–18] which suggests that environmental precipitating factors are likely to be varied in different parts of the world, explaining the differential presentation of HLA-B*07 and HLA-B*40 in these groups of patients.

## Figures and Tables

**Table 1:** Distribution of HLA-B alleles among B27 negative SpA patients and controls from Western India (patients (*n* = 100); controls (*n* = 100)).

Serial number	HLA-B	%PF	%P.F	OR	95% CI	*χ* ^2^Y	*P* value
1	B*51	10	13	0.743	0.309–1.785	0.196	0.506
2	B*52	7	8	0.865	0.301–2.485	0.072	0.788
3	B*07	**54**	**18**	**5.348**	**2.808–10.186**	**26.584**	**1.14*E *− 07**
4	B*15	19	12	1.720	0.785–3.765	1.374	0.171
5	B*08	12	14	0.837	0.366–1.914	0.044	0.674
6	B*18	6	9	0.645	0.220–1.887	0.288	0.420
7	B*13	4	3	1.347	0.293–6.183	0.148	0.700
8	B*40	**17**	**32**	**0.435**	**0.222–0.850**	**5.298**	**0.013**
9	B*35	33	44	0.626	0.353–1.113	2.112	0.109
10	B*57	9	7	1.314	0.469–3.678	0.067	0.602
11	B*58	8	4	2.080	0.607–7.169	0.797	0.233
12	B*44	9	15	0.560	0.232–1.348	1.184	0.191
13	B*47	3	6	0.484	0.117–1.995	0.465	0.306
14	B*48	2	0	5.102	0.241–107.70	0.505	0.155
15	B*53	1	1	1.000	0.061–16.224	0.000	1.000
16	B*49	1	3	0.326	0.033–3.196	0.255	0.312
17	B*38	0	1	0.330	0.013–8.205	1.005	0.316
18	B*78	0	2	0.196	0.009–4.138	0.505	0.155
19	B*81	1	3	0.326	0.333–3.196	0.255	0.312
20	B*37	1	3	0.326	0.333–3.196	0.255	0.312
21	B*42	0	1	0.330	0.013–8.205	1.005	0.316
22	B*56	3	1	3.062	0.312–29.965	0.255	0.312

N+: number positive, PF: phenotype frequency, OR: odds ratio, *χ* ^2^Y: chi-square with Yates' correction.

**Table 2:** Clinical presentation of B27 negative SpA patients.

Serial number	Character	B27 negative *n* = 100	Normal controls *n* = 100
1	Age of onset	%	%
	≤15	11	14
	16–30	52	21
	31–45	30	58
	>45	7	7
2	Sex:		
	M	80	56
	F	20	44
3	Fam H/O arthritis	13	0
4	Polyarticular arthritis >3 Jts	40	0
5	Pauciarticular	35	0
6	Monoarticular	25	0
7	Chest exp ≤5 cm	25	0
8	Spine flx ≤5 cm	50	0
9	Tend SI JT	25	0
10	Knee jt	54	0
11	Ankle jt	30	0
12	L-spine	38	0
13	C-spine	43	0
14	Hip jt	46	0
15	SI jt	25	0
16	Shoulder jt	7	0
17	Wrist jt	11	0
18	Foot jt	7	0
19	Hand jt	9	0
20	Enthesitis	84	0
